# Severity of Fatigue and Its Relationship with TSH before and after Levothyroxine Replacement Therapy in Patients with Primary Hypothyroidism

**DOI:** 10.3390/biomedicines11030811

**Published:** 2023-03-07

**Authors:** María Guadalupe Ruíz-Pacheco, Irma Hernández, Guadalupe Hernández-Estrella, Lourdes Basurto, Guadalupe Vargas-Ortega, Baldomero González-Virla, Mario Molina-Ayala, Alex Francisco Hernández-Martínez, Rosamaría Luengas-Mondragón, Angel Alejandro Hernández-Allende, Victoria Mendoza-Zubieta, Lourdes Balcázar-Hernández

**Affiliations:** 1 Centro Médico Nacional Siglo XXI, Department of Endocrinology, Hospital de Especialidades, Instituto Mexicano del Seguro Social, Mexico City 06720, Mexico; 2 Faculty of Medicine, Universidad Nacional Autónoma de México, Mexico City 04510, Mexico; 3 Unidad de Medicina Familiar No. 69, Department of Family Medicine, Instituto Mexicano del Seguro Social, Calle José María Morelos 210-232, Centro, Texcoco 56100, Mexico; 4 Endocrine Diseases Research Unit, Centro Médico Nacional Siglo XXI, Instituto Mexicano del Seguro Social, Mexico City 06720, Mexico

**Keywords:** fatigue, hypothyroidism, thyrotropin, levothyroxine

## Abstract

Background: Fatigue is a common symptom in hypothyroidism; however, the effect of levothyroxine on fatigue has been little studied. The aim of this study was to evaluate the effect of levothyroxine on fatigue in Latino patients with primary hypothyroidism, as well as the association of TSH and free T4 (FT4) with the severity and persistence of fatigue. Methods: A prospective study was performed in 92 patients with primary hypothyroidism. Fatigue severity scale (FSS) scores and clinical and biochemical characteristics before and at 6 months of levothyroxine were evaluated. Results: After 6 months of levothyroxine, a reduction in FSS (53 (47–57) vs. 36 (16–38); *p* = 0.001) and fatigue frequency (45.7% vs. 26.1%; *p* = 0.008) was evident. Both before and after 6 months of levothyroxine, there was a positive correlation of the FSS score with TSH and a negative correlation with FT4. Persistent fatigue was associated with a pretreatment FSS score (r = 0.75; *p* = 0.001) and diabetes (r = 0.40; *p* = 0.001). An FSS > 34 (RR 3.9 (95% CI 1.43–10.73; *p* = 0.008)), an FSS > 36 (RR 3.23 (95% CI 1.21–8.6; *p* = 0.019)), and diabetes (RR 5.7 (95% CI 1.25–9.6; *p* = 0.024)) before treatment were risk factors for persistent fatigue. Conclusions: Levothyroxine improved fatigue in most patients. Diabetes and an FSS score >34 or >36 before treatment were risk factors for persistent fatigue.

## 1. Introduction

Fatigue is defined as “a sense of physical tiredness and lack of energy, distinct from sadness or weakness” [1]. The most recent definitions refer to fatigue as a suboptimal psychophysiological condition caused by exertion; this state causes changes that may reduce mental processing or physical activity [2]. Fatigue is related with alterations in muscle activity, proprioception, and cognitive function [3], and has been associated with chronic diseases such as hypothyroidism [4]. It has been reported that up to one-third of patients presenting with fatigue have thyroid disease, predominantly in women [5]. In addition, the frequency of fatigue is higher among patients with autoimmune thyroid disease compared to patients with post-thyroidectomy hypothyroidism due to differentiated thyroid cancer [6].

A previous study demonstrated that hormone replacement with levothyroxine is associated with improvement of fatigue in most patients with hypothyroidism, mainly in those with post-ablation hypothyroidism, proposing a possible relationship between thyrotropin (TSH) levels and fatigue [7]. Despite these data, there is no information available about the effect of levothyroxine on fatigue in the Latino population with primary hypothyroidism. The aim of our study was to evaluate the effect of levothyroxine replacement therapy on fatigue in Latino patients with primary hypothyroidism (autoimmune and unspecified hypothyroidism), the association of TSH and free T4 (FT4) with fatigue severity scale (FSS) score and the score related to the persistence of fatigue despite levothyroxine replacement therapy.

## 2. Materials and Methods

A prospective, longitudinal, clinical study in Latino patients with primary hypothyroidism due to autoimmune and unspecified etiology was conducted in a tertiary healthcare center. Patients with other causes of hypothyroidism such as iodine deficiency, post-ablative, post-thyroidectomy, and central hypothyroidism were excluded to avoid the effect of other diseases related to acquired hypothyroidism that could cause or worsen fatigue. Non-probabilistic sampling was performed, without gender preference.

Demographics and clinical characteristics, including the comorbidities (diabetes, dislipidemia, obesity, hypertension, rheumatologic/autoimmune diseases), were evaluated. All patients had adequate control of comorbidities. Clinical, fatigue severity, and biochemical characteristics of patients were evaluated both before and at 6 months after levothyroxine replacement therapy.

Levothyroxine replacement therapy: Patients were treated with L-thyroxine monotherapy at an initial dose of 1.6 μg/kg according to international recommendations. Dose adjustments were guided by serum TSH determinations between 4 and 8 weeks after initiation of therapy, with dose adjustments until normalization of the thyroid profile (TSH and FT4). According to the recommendations, patients with subclinical hypothyroidism were treated when TSH values were higher than 10 mIU/L, when elevated TSH levels were found accompanied by symptoms and positive antithyroid antibodies, as well as in women seeking pregnancy [8,9]. No patient was pregnant during the study. All patients included had a normal thyroid profile at 6 months after the beginning of levothyroxine replacement therapy.

Fatigue severity evaluation: The fatigue severity scale (FSS) was used to evaluate fatigue both before and at 6 months after the beginning of levothyroxine replacement therapy. The FSS is one of the most commonly used fatigue questionnaires in chronic diseases [10] and is validated in our population [11]. The FSS consists of nine items that measure how fatigue affects motivation, exercise, physical functioning, carrying out duties, work, family, or social life. The scores are averaged across the nine statements and a score ≥36 indicates a clinically significant trait level of fatigue [10,11]. The delta of FSS (ΔFSS) was the difference between the FSS after treatment and the FSS before treatment.

Biochemical and Hormonal Measurements: The thyroid profile included the measurement of TSH and FT4, and was evaluated before and after 6 months of levothyroxine replacement therapy. The electrochemiluminescence immunoassay “ECLIA” (Roche Diagnostics, Roche^®^, Indianapolis, IN, USA) was used to measurement TSH in serum, with a sensitivity of 0.014 μIU/mL, inter- and intra-assay coefficients of variation (CV) <10%, respectively, and a normal reference range of 0.270 to 4.20 μIU/mL. FT4 was measured by an electrochemiluminescence immunoassay “ECLIA” (Roche Diagnostics, Roche^®^), with a sensitivity of 0.02 ng/dL, inter- and intra-assay coefficients of variation (CV) ≤30%, respectively, and a normal reference range of 0.93 to 1.7 ng/dL. Thyroid peroxidase antibodies (TPOAb) (normal reference range <34 IU/mL) and thyroglobulin antibodies (TgAb) (normal reference range <1.8 IU/mL) were assessed by enzyme-linked immunosorbent assay.

Statistical Analysis: All quantitative variables were tested using non-parametric tests and described as medians with interquartile range (IQR). Proportions (expected frequency, prevalence) were used for the qualitative variables. For quantitative variables, the Wilcoxon test was used to compare two paired samples, and the Mann–Whitney U test was used to compare differences between two independent groups. For qualitative variables, we used the Fisher test. An ROC curve was designed to predict the best pretreatment FSS score cut-off point for predicting persistent fatigue. Spearman’s rank correlation coefficient was used to calculate the correlation between the variables. A multivariate, stepwise logistic regression analysis was carried out to explore which variables were associated with persistent fatigue. A *p* < 0.05 was considered as statistically significant. Statistical software consisted of SPSS (v.24, IBM^®^, Armonk, NY, USA) and STATA (v.16.1., StataCorp^®^, College Station, TX, USA).

Ethics: The study protocol was approved by our local ethics and scientific committees and all patients signed the appropriate informed consent.

## 3. Results

### 3.1. Baseline Characteristics of the Population

A total of 92 patients with primary hypothyroidism were included; 94.6% (n = 87) were women. The median age was 32 (IQR 31–41) years. The baseline characteristics of patients are included in Table 1.

### 3.2. Fatigue Severity Scale before and at 6 Months of Levothyroxine Replacement Therapy

After 6 months of levothyroxine replacement therapy, there was a reduction in TSH levels (52.4 (25–100) vs. 2.3 (1.7–3.5) mIU/L; *p* = 0.002), an increase in FT4 (0.38 (0.14–1.12) vs. 1.04 (0.99–1.15) pg/mL; *p* = 0.007), and a reduction in the score given to each of the FSS items and in the total FSS score (Table 2) compared to their pretreatment characteristics. The median dose of levothyroxine was 100 mg per day (IQR 25–150). All patients achieved the normalization of the thyroid profile.

According to the FSS score, a decrease in the frequency of fatigue was evident after 6 months of levothyroxine replacement therapy compared to its frequency before treatment (45.7% (n = 42) vs. 26.1% (n = 24); *p* = 0.008). The ΔFSS was 17 (31–19). There was no association between the ΔFSS and the levothyroxine dose (r = 0.14; *p* = 0.25)

### 3.3. TSH and Free T4 According to the FSS Score after Levothyroxine Replacement Therapy

After 6 months of levothyroxine replacement therapy, patients with persistent fatigue, according to an FSS score greater than 36, had a higher pretreatment FSS score (48 (IQR 33–50) vs. 27 (IQR 16–48); *p* = 0.003) and higher pretreatment TSH (31 (IQR 6.8–31.9) vs. 8 (IQR 1.3–8.7) mIU/L; *p* = 0.001) compared to those with an FSS score less than 36. There were no differences in FT4 (*p* = 0.80).

### 3.4. FSS Score According to the Etiology and Presentation of Primary Hypothyroidism

According to the presentation of primary hypothyroidism, there were differences in pretreatment TSH and FT4, as well as in the doses of levothyroxine required for normalization of the thyroid profile in patients with overt hypothyroidism compared to subclinical hypothyroidism (Table 3). There was no difference in the FSS score both before and at 6 months of levothyroxine replacement therapy.

### 3.5. FSS Score between Autoimmune Hypothyroidism and Unspecified Hypothyroidism

Patients with autoimmune hypothyroidism had lower FT4 before levothyroxine and higher TPOAb and TgAb compared with patients with unspecified hypothyroidism. There was no difference in TSH and the FSS score before and after levothyroxine replacement therapy (Table 4). There was no association between the FSS score and the presence of autoimmune disease (r = 0.15; *p* = 0.84), TPOAb (r = 0.34; *p* = 0.43), or TgAb (r = 0.29; *p* = 0.37).

In a subanalysis of patients with autoimmune hypothyroidism (n = 51), normalization of TSH and FT4, as well as improvement of the FSS score after treatment was evidenced (Table 5). There were no differences in TPOAb and TgAb. Before levothyroxine, the FSS score was negatively associated with FT4 (r = −0.92; *p* = 0.001) and positively associated with TSH (r = 0.30; *p* = 0.03). After 6 months of levothyroxine, the FSS score was associated with TSH (r = 0.67; *p* = 0.001). There was no association between the FSS score and TPOAb or TgAb either before or after levothyroxine.

### 3.6. Association between FSS, TSH and FT4 before and at 6 Months of Levothyroxine Replacement Therapy

Prior to administration of levothyroxine, a positive correlation of the FSS score with TSH (r = 0.45; *p* = 0.001; linear regression β = 0.25: *p* = 0.036) and a negative correlation with FT4 (r = −0.39; *p* = 0.001; linear regression β = −0.080: *p* = 0.05) were evident.

After 6 months of levothyroxine replacement therapy, the positive correlation of the FSS score with TSH (r = 0.34; *p* = 0.001; linear regression β = 0.34: *p* = 0.021) and the negative correlation with FT4 (r = −0.35; *p* = 0.04; linear regression β = −0.15: *p* = 0.19) were constant.

The persistence of fatigue was positively associated with TSH before treatment (r = 0.41; *p* = 0.04), the FSS score before treatment (r = 0.75; *p* = 0.001), the FSS score after treatment (r = 0.30; *p* = 0.003), diabetes (r = 0.40; *p* = 0.001), hypertension (r = 0.24; *p* = 0.02), and fibromyalgia (r = 0.36; *p* = 0.001).

### 3.7. Risk Factors for the Persistence of Fatigue at 6 Months of Levothyroxine Replacement Therapy

An FSS > 34 before levothyroxine (AUC 0.72, 95%CI 0.58–0.81) had a sensitivity of 70% and a specificity of 62% to predict fatigue at 6 months of levothyroxine.

A multiple regression model adjusted by the comorbidities (diabetes, hypertension, obesity, rheumatoid arthritis, and fibromyalgia), the FSS score cut-off of >34 and the traditional cut-off of >36 before levothyroxine showed that diabetes (RR 5.7 (95%CI 1.25–9.6; *p* = 0.024)), the FSS > 34 pre-treatment (RR 3.9 (95%CI 1.43–10.73; *p* = 0.008)), as well as the FSS score cut-off of >36 (RR 3.23 (95%CI 1.21–8.6; *p* = 0.019)), were risk factors for persistent fatigue at 6 months of levothyroxine replacement therapy (Table 6).

## 4. Discussion

This study demonstrates that levothyroxine replacement therapy improved fatigue (according to the FSS score) in most patients with primary hypothyroidism, and this score was positively associated with TSH and negatively associated with FT4 both before and after treatment. In addition, diabetes and an FSS score >34 before, as was the traditional cut-off point of an FSS score >36 before levothyroxine, were risk factors for persistent fatigue at 6 months of levothyroxine replacement therapy.

There is a wide spectrum of symptoms associated with hypothyroidism, and some may be nonspecific. Fatigue is one of the most common symptoms, regardless of the presentation of hypothyroidism (subclinical or overt hypothyroidism) [12]. Fatigue is a non-specific symptom; therefore, it is convenient to evaluate it objectively through different instruments, such as the FSS. In the context of fatigue and hypothyroidism, the associations with the etiology or type of presentation of hypothyroidism have been proposed.

Regarding disease etiology, one study demonstrated that fatigue and fatigue-related symptoms in hypothyroidism were more pronounced in patients with autoimmune diseases compared to patients with differentiated thyroid carcinoma, which was reflected in significantly higher scores on the MFI-20 questionnaire; however, these results could not be associated with thyroid hormone parameters [6]. Autoimmunity has been shown to have an impact on the quality of life of patients with autoimmune hypothyroidism, especially in terms of psychological symptoms; however, its direct association with fatigue has been unclear [13]. Despite these data, in our study, no differences in the FSS score were observed when comparing patients with autoimmune hypothyroidism versus unspecified hypothyroidism; similarly, no association was found between the fatigue severity scale score and the presence of autoimmune disease or TPOAb and TgAb levels. Specifically in our population of patients with autoimmune hypothyroidism, we only found association of the FSS score with TSH and FT4, with no association with TPOAb or TgAb. On the other hand, there was no difference in the clinical parameters or the FSS score when comparing patients with subclinical hypothyroidism and overt hypothyroidism, both before and after levothyroxine replacement therapy. Despite these findings, the greater decrease in the FSS score in patients with overt hypothyroidism (15 points) compared to those with subclinical hypothyroidism (7 points) is striking. This result, although not statistically significant, may be relevant in clinical practice, highlighting the importance of levothyroxine treatment in patients with subclinical hypothyroidism for the improvement of fatigue.

One of the expectations of both the physician and the patient when initiating treatment with levothyroxine is the improvement of symptoms related to hypothyroidism; however, the results of this goal are heterogeneous according to clinical studies. In a previous study, Watt, et al. used the ThyPRO questionnaire to measure a number of aspects of quality of life relevant to patients with benign thyroid disease. According to this instrument, there was no significant change in hypothyroid symptoms after 6 months of levothyroxine replacement therapy, including those related to fatigue [13]. One study evidenced that TSH level reduction was associated with fatigue relief; however, it was unclear whether fatigue relief was associated with the magnitude of TSH reduction or with absolute TSH levels after levothyroxine replacement therapy [7]. In patients with subclinical hypothyroidism, it has been reported that levothyroxine did not improve fatigue-related symptoms [14], and physical or mental fatigability [15] compared to the placebo, even a meta-analysis corroborated that levothyroxine was not associated with improvements in overall quality of life or hypothyroidism-related symptoms in patients with subclinical hypothyroidism [16]. In our study, we evidenced improvement in the FSS score after levothyroxine replacement therapy in most patients. Additionally, we found that absolute TSH and FT4 levels were associated with the FSS score, corroborating a relationship between the magnitude of fatigue, hypothyroxinemia, and hyperthyrotropinemia.

Our study showed that hypothyroidism could be one of multiple factors contributing to fatigue in these patients, and levothyroxine replacement therapy could improve fatigue in most of them. However, about a quarter persisted with fatigue despite normalization of the thyroid profile, implying that other contributing factors should be sought, with levothyroxine treatment being only one point of intervention among a sea of possibilities. According to comorbidities, we found that diabetes is a risk factor for persistent fatigue after levothyroxine replacement therapy, even though these patients had adequate glycemic control. Fatigue is a common symptom in diabetes, usually related to hyperglycemia. The prevalence of fatigue in people living with diabetes remains unclear due to the lack of a cut-off point for the instrument, and factors associated with fatigue such as BMI and glycosylated hemoglobin have been proposed, but the data are still inconclusive [17,18]. This demonstrates the importance of a comprehensive approach and treatment of both hypothyroidism and comorbidities.

It has been reported that 10–15% of patients persist with hypothyroidism-related symptoms despite treatment, mainly with impaired quality of life and mood disorders [19,20,21]. Studies focused on fatigue have been scarce so far; therefore, it is difficult to contrast the proportion of persistent fatigue found in our study. However, if we compared it with the reported proportion of persistence of all hypothyroidism-related symptoms, the one found in this study was higher (26.1 vs. 10–15%), which may be related to a considerable proportion of patients with comorbidities in our population that may perpetuate fatigue, mainly diabetes, which was a risk factor for persistent fatigue. The persistence of symptoms related to hypothyroidism may be related to inadequate levothyroxine replacement with the resulting persistence of elevated TSH and/or hypothyroxinemia [22]; however, the persistence of symptoms can also occur in the context of normalization of the thyroid profile, as in our study. In this study, we did not find an association between the ΔFSS and the levothyroxine dose. Some cross-sectional studies have reported lower quality of life in patients with hypothyroidism and normal thyrotropin levels [22,23,24,25,26], including the reduction of vitality [26].

Some potential causes of residual symptoms in patients with normal thyrotropin values have been proposed, such as the presence of symptoms caused by accompanying conditions or comorbidities, incorrect attribution and unrealistic patient expectations, awareness of chronic condition, tissue hypothyroidism (low T3 levels at the tissue or cellular level), and alterations in the conversion of T4 to T3 or in the entry of thyroid hormone into cells [19]. There is no consensus about the best treatment approach for those patients who do not respond to standard treatment with levothyroxine alone. According to the recommendations, patients with hypothyroidism should be treated with levothyroxine monotherapy [8,9]; however, the addition of liothyronine has been proposed as a beneficial and safe therapy in the management of hypothyroidism symptoms, mainly in quality of life [20,21,27], weight management, fatigue, mood, and memory [28]. The development of clinical trials evaluating the effect of this intervention on persistent fatigue in different populations would be encouraging in the management of patients with hypothyroidism. Other strategies such as aromatherapy [29]; dietary intervention of green vegetables, beef, whole milk, and butter [30]; and yoga [31] have been associated with a reduction in fatigue in patients with hypothyroidism.

Among the strengths of the study are the exploration of a little-known issue in the Latino population with hypothyroidism, the use of a standardized scale that evaluates the presence and intensity of fatigue, the evaluation of the differences of this score according to the etiology, and presentation of hypothyroidism, as well as the evaluation of the association of the FSS score with biochemical parameters. Limitations of the study include a relatively small sample size due to the inclusion of patients only with primary autoimmune hypothyroidism or of unspecified cause, the lack of a control group, a short follow-up, as well as the absence of intervention with other strategies such as liothyronine in those with persistent fatigue. Another limitation was the majority inclusion of women despite non-random sampling, which could have a gender effect on the results, but could not be assessed due to the small number of men included. The inclusion of a greater number of women may be related to the 6- to 9-fold increased risk of hypothyroidism in women compared to men. Some of the factors that have been proposed are pregnancy, postpartum, menopause, and aging [8,32,33]. The development of future clinical studies that include patients with other causes of hypothyroidism, the evaluation of other associated factors for persistent fatigue, as well as the use of combination therapy with liothyronine, will allow overcoming these limitations and enrich the knowledge about fatigue in patients with hypothyroidism in order to improve their prognosis.

## 5. Conclusions

Levothyroxine replacement therapy improved fatigue in patients with primary hypothyroidism according to the FSS score, and this score was positively associated with TSH and negatively associated with FT4, both before and after treatment. Persistent fatigue was associated with a pretreatment FSS score and diabetes. The presence of diabetes, an FSS score >34, as well as the traditional FSS score cut-off point >36 before levothyroxine replacement therapy were risk factors for persistent fatigue at 6 months of treatment.

## Figures and Tables

**Table 1:** Baseline characteristics of the participants in the study (n = 92).

Variable	Result
Age (years)	32 (31–41)
Sex; n (%)	Women: 87 (94.6) Men: 5 (5.4)
BMI (kg/m^2^)	27.1 (25.3–33.4)
Presentation of hypothyroidism; n (%)	Subclinical hypothyroidism: 28 (30.4) Overt hypothyroidism: 64 (69.6)
Type of primary hypothyroidism; n (%)	Autoimmune hypothyroidism: 51 (55) Unspecified hypothyroidism: 41 (45)
Time from onset of symptoms to diagnosis (months)	4 (3–36)
Clinical manifestations; n (%)	Apparently asymptomatic: 30 (32.6) Symptomatic: 62 (67.4) Asthenia: 58 (63) Muscular pain: 4 (4.3) Gastrointestinal symptoms: 9 (9.8) Dry skin, hypohidrosis: 12 (13) Bradypsychia: 5 (5.4)
Comorbidities; n (%)	Diabetes: 20 (21.7) Hypertension: 32 (34.8) Obesity: 26 (28.3) Dyslipidemia: 18 (19.6) Rheumatoid arthritis: 6 (6.5) Fibromyalgia: 4 (4.3)

Quantitative variables were reported as median and interquartile range. BMI: body mass index.

**Table 2:** Fatigue severity scale before and after 6 months of levothyroxine replacement therapy.

Item	Before Levothyroxine	After 6 Months of Levothyroxine	*p* Value
1. My motivation is lower when I am fatigued	7 (6–7)	4 (3–5)	0.001 *
2. Exercise brings on my fatigue	7 (2–7)	4 (1–5)	0.004 *
3. I am easily fatigued	6 (5–7)	4 (1–5)	0.002 *
4. Fatigue interferes with my physical functioning	6 (5–7)	4 (1–5)	0.001 *
5. Fatigue causes frequent problems for me	6 (5–7)	4 (1–6)	0.007 *
6. My fatigue prevents sustained physical functioning	7 (5–7)	3 (1–5)	0.001 *
7. Fatigue interferes with carrying out certain duties and responsibilities	7 (5–7)	3 (2–5)	0.001 *
8. Fatigue is among my three most disabling symptoms	7 (6–7)	4 (2–6)	0.001 *
9. Fatigue interferes with my work, family, or social life	3 (1–5)	1 (1–3)	0.001 *
Total	53 (47–57)	36 (16–38)	0.001 *

Quantitative variables were reported as median and interquartile range. * *p* > 0.05 = statistical significance.

**Table 3:** Clinical and biochemical differences between subclinical and overt hypothyroidism.

	Subclinical Hypothyroidism (n = 28)	Overt Hypothyroidism (n = 64)	*p* Value
Age (years)	51 (30–68)	52 (41–63)	0.61
BMI (kg/m^2^)	26.3 (24.2–30.4)	25.9 (24.3–31.2)	0.99
Doses of levothyroxine for thyroid profile normalization (mg/day)	68 (50–100)	100 (75–150)	0.006 *
TSH before therapy (mIU/L)	12.2 (9.8–43.5)	47.5 (10.9–74.9)	0.001 *
TSH at treatment (mIU/L)	1.07 (0.9–3.2)	1.3 (1.0–3.4)	0.12
FT4 before therapy (pg/mL)	1.3 (0.9–1.6)	0.52 (0.15–0.71)	0.02 *
FT4 at treatment (pg/mL)	1.01 (0.94–1.2)	1.1 (0.99–1.19)	0.88
FSS score before levothyroxine	31 (26–45)	33 (16–51)	0.99
FSS score at treatment	24 (20–33)	18 (11–36)	0.37

Quantitative variables were reported as median and interquartile range. BMI: body mass index; TSH: thyroid stimulating hormone; FT4: free thyroxine; FSS: fatigue severity scale. * *p* > 0.05 = statistical significance.

**Table 4:** Clinical and biochemical differences between autoimmune and unspecified hypothyroidism.

	Autoimmune (n = 51)	Unspecified Hypothyroidism (n = 41)	*p* Value
Age (years)	32 (31–41)	37 (32–50)	0.36
BMI (kg/m^2^)	27.1 (25.3–33)	26.9 (24.1–31.4)	0.86
Doses of levothyroxine for thyroid profile normalization (mg/day)	100 (50–125)	75 (50–150)	0.07
TSH before therapy (mIU/L)	19.8 (7.6–43.5)	17.9 (8.9–52.9)	0.26
TSH at treatment (mIU/L)	1.8 (1.3–3.9)	1.65 (1.0–3.6)	0.13
FT4 before therapy (pg/mL)	0.16 (0.14–0.52)	0.64 (0.32–1.2)	0.02 *
FT4 at treatment (pg/mL)	1.01 (0.94–1.2)	1.1 (0.99–1.19)	0.47
Thyroid Peroxidase Antibodies (IU/mL)	82 (43–93)	5.8 (3.2–30.1)	0.001 *
Thyroglobulin antibodies (IU/mL)	25 (8.9–36.7)	0.5 (0.3–1.4)	0.003 *
FSS score before levothyroxine	32 (15–48)	31 (30–47)	0.15
FSS score at treatment	24 (16–32)	21 (18–29)	0.09

Quantitative variables were reported as median and interquartile range. BMI: body mass index; TSH: thyroid stimulating hormone; FT4: free thyroxine; FSS: fatigue severity scale. * *p* > 0.05 = statistical significance.

**Table 5:** Biochemical characteristics of patients with autoimmune hypothyroidism before and after 6 months of levothyroxine replacement therapy.

	Before Levothyroxine	After 6 Months of Levothyroxine	*p* Value
TSH (mIU/L)	19.8 (7.6–43.5)	1.8 (1.3–3.9)	0.002 *
FT4 (pg/mL)	0.16 (0.14–0.52)	1.01 (0.94–1.2)	0.01 *
Thyroid Peroxidase Antibodies (IU/mL)	82 (43–93)	78 (38–101)	0.35
Thyroglobulin antibodies (IU/mL)	25 (8.9–36.7)	20.7 (9.3–34.1)	0.15
FSS score	32 (15–48)	24 (16–32)	0.016

Quantitative variables were reported as median and interquartile range. BMI: body mass index; TSH: thyroid stimulating hormone; FT4: free thyroxine; FSS: fatigue severity scale. * *p* > 0.05 = statistical significance.

**Table 6:** Risk factors associated with persistence of fatigue after 6 months of levothyroxine replacement therapy.

	Relative Risk	95% Confidence Interval	*p* Value
FSS > 34	3.9	1.43–10.73	0.008 *
FSS > 36	3.2	1.21–8.6	0.019 *
Diabetes	5.7	1.25–9.6	0.024 *
Hypertension	1.1	0.25–4.7	0.89
Obesity	1.16	0.20–1.27	0.22
Rheumatoid arthritis	1.2	0.1–47	0.78
Fibromyalgia	0.9	0.3–28	0.82

FSS: fatigue severity scale. * *p* > 0.05 = statistical significance.

## Data Availability

The data will not be made public due to ethical regulations.
